# Comparative Analysis of Structural and Functional Properties of Dietary Fiber from Four Grape Varieties

**DOI:** 10.3390/molecules29112619

**Published:** 2024-06-02

**Authors:** Yingying Chang, Ran An, Sijie Sun, Min Hou, Fuliang Han, Shiren Song

**Affiliations:** 1 School of Agriculture and Biology, Shanghai Jiao Tong University, Shanghai 200240, China; 18438615801@163.com (Y.C.); ran.an@sjtu.edu.cn (R.A.); sun_sijie@126.com (S.S.); 2 School of Public Health, Shanghai Jiao Tong University School of Medicine, Shanghai 200240, China; houmin@sjtu.edu.cn; 3 School of Wine, Northwest A&F University, Yangling, Xianyang 712100, China

**Keywords:** Muscadine grapes, dietary fiber, structural characterization, physicochemical properties

## Abstract

Muscadine grapes are characterized by their large and abundant seeds and hard and thick skins that contain significant amounts of dietary fiber (DF). The current study investigated the chemical constituents, molecular architecture, and physicochemical attributes of DF derived from Muscadine grapes (Granny Val and Alachua) and compared them with those derived from Shine Muscat and Kyoho. Using a combined enzymatic method, the total dietary fiber (TDF) was extracted and divided into two parts: soluble dietary fiber (SDF) and insoluble dietary fiber (IDF). TDF (mainly IDF, with a small fraction of SDF) was dominated by cellulose, followed by pectin and hemicellulose. In addition, Granny Val and Alachua had a significantly higher abundance of TDF and IDF compared with Shine Muscat and Kyoho. Moreover, Shine Muscat had significantly the highest abundance of SDF among the four grape varieties. Of note, IDF from Granny Val and Alachua exhibited a complex and dense texture on its surface, and notably outperformed Shine Muscat and Kyoho in terms of cholesterol, fatty acid, heavy metal adsorption, and antioxidant activity. Collectively, Muscadine grapes, i.e., Granny Val and Alachua in the current study, possessed elevated DF levels (predominantly IDF), and their enhanced bioactivity underscored their potential as a potential food ingredient for further use.

## 1. Introduction

Muscadine grapes (*Muscadinia rotundifolia Michx*.) belong to the *Vitaceae* family and the *Muscadine* genus [1], native fruit crops of the southeastern United States [2]. These grapes can thrive in high-temperature and high-humidity environments, demonstrating resistance or even immunity to various pests and diseases, such as grape downy mildew, root aphids, Pierce’s disease, and root-knot nematodes [3]. In terms of processing applications, Muscadine grapes are versatile, suitable for winemaking, drying, sauce preparation, and juicing [4]. Their high health and nutritional values are considered beneficial for humans, attributed to their rich content in polyphenols, trace elements, and vitamin C. Notably, they are abundant in polyphenolic substances such as tannic acid, flavonol, resveratrol, and proanthocyanidins [5,6]. These compounds exhibit various physiological actions, including antioxidation, antibacterial, anticancer, and anti-inflammatory properties [7,8,9,10]. Among all grapes, only those from the Muscadine genus contain tannic acid-type polyphenols [11]. However, most research on Muscadine grapes has focused on polyphenols and other bioactive substances, leaving a gap in dietary fiber (DF) characteristics.

DF refers to edible carbohydrate polymers composed of no less than three monomers that are not hydrolyzed or absorbed by endogenous digestive enzymes in the small intestine [12]. Based on their solubility, DFs can be classified into IDFs (hemicellulose, cellulose, lignin, etc.) and soluble dietary fibers (SDFs) (mucilage, pectin, gum, and beta-glucan) [13,14]. In recent years, DF has garnered increased attention for its potential health promoting effects. Experimental and epidemiological studies have demonstrated its involvement in various physiological activities, such as reducing the incidence of cardiovascular diseases, diabetes, colorectal cancer, and obesity [15,16,17]. Furthermore, DF, as a natural food additive, not only enhances the quality of foods but also improves the flavor of processed foods and extends product shelf life [18,19]. Nevertheless, studies investigating the composition and functional properties of IDF and SDF in Muscadine grapes are still lacking.

Previous studies have revealed that grape DF is mostly derived from grape skin, seed, and pulp [20], collectively accounting for 50–75% of its dry weight [21]. Available research on grape dietary fibers predominantly focuses on the following varieties: Manto Negro, Pinot Noir, Cabernet Sauvignon, Merlot, Chardonnay, Syrah, Prensal Blanc, and Tannat [22,23,24]. Nevertheless, studies related to Muscadine grapes primarily focus on their polyphenolic activity [5,6]. In addition to polyphenolic substances [22], Muscadine grapes are characterized by their large and abundant seeds and hard and thick skins [25], which contain significant amounts of DF. However, limited studies have specifically investigated the dietary fiber of Muscadine grapes. This study aims to fill this gap by conducting a comparative study of structural, physicochemical, and functional properties of DF from Muscadine grapes (Alachua and Granny Val grapes) and from Shine Muscat and Kyoho grapes. We hypothesize that DFs derived from different grape varieties exhibit variations in their structural, physicochemical, and functional properties.

## 2. Results

### 2.1. Proximate Composition Analysis

**Figure 1:**
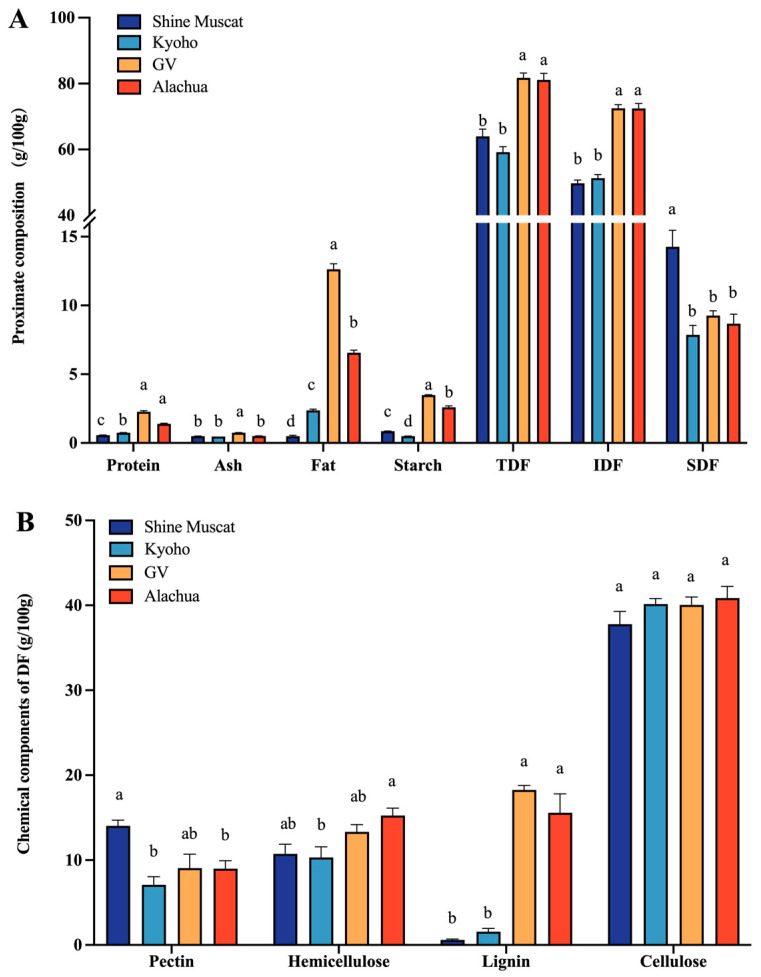
Proximate composition of four grape varieties. (**A**) Proximate chemical composition of different grape powders. (**B**) Main component of DF in four grape varieties. TDF—total dietary fiber. IDF—insoluble dietary fiber. SDF—soluble dietary fiber; a,b,c,d—different small letters indicate significant difference.

In all studied grape varieties, the constituted primary component was predominantly composed of IDF, with a minor proportion of SDF, followed by fat, starch, and protein (Figure 1A). The protein and starch content in the four grape varieties was below 5 g per 100 g dry grapes. The fat content was highest in Granny Val, followed by Alachua, Kyoho, and Shine Muscat. Among the four grape varieties, the abundance of TDF and IDF in Granny Val and Alachua was significantly higher than in Shine Muscat and Kyoho. Shine Muscat had a significantly higher abundance of SDF compared with the other grapes.

The crude grape powder was separated into pectin, hemicellulose, lignin, and cellulose (Figure 1B). Among these polysaccharides, cellulose was of the highest abundance. Remarkably, the lignin content in Granny Val and Alachua did not differ significantly from one another but was significantly higher than in Kyoho and Shine Muscat. The content of pectin was significantly higher in Shine Muscat (14.04 ± 0.66 g/100 g) compared with the other grapes. Concerning the content of hemicellulose, it was lowest in Kyoho, while no significant difference was identified among the other grapes. Collectively, the Muscadine grapes, i.e., Alachua and Granny Val, demonstrated the characteristic of high lignin content compared with the conventional table grapes, i.e., Kyoho and Shine Muscat, in the current study.

### 2.2. Structural Properties

#### 2.2.1. Monosaccharide Composition

The monosaccharide composition of SDF and IDF differed per grape variety (Table 1). Specifically, in the four grape varieties, the top two monosaccharides within IDF were glucose and xylose. Among IDF in the four grape varieties, IDF in Shine Muscat had the highest glucose content, while IDF in Alachua had the highest content of xylose. Different to IDF, galacturonic acid, glucose, galactose, and arabinose dominated the SDF counterpart. Glucose dominated the SDF in Kyoho, while galacturonic acid dominated the SDF in the other grapes.

#### 2.2.2. SEM Analysis

**Figure 2:**
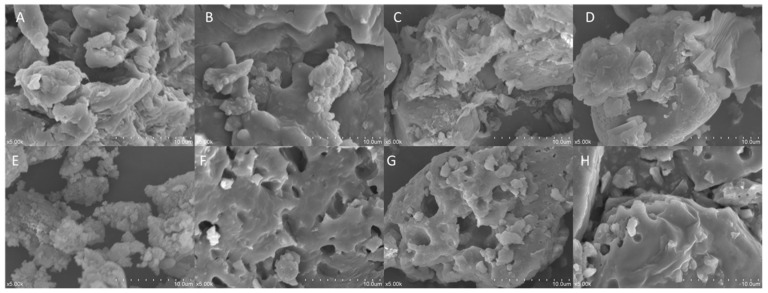
Scanning electron microscopy images (magnification 500×) of IDF from (**A**) Shine Muscat, (**B**) Kyoho, (**C**) Granny Val, and (**D**) Alachua; and SDF from (**E**) Shine Muscat, (**F**) Kyoho, (**G**) Granny Val, and (**H**) Alachua. IDF—insoluble dietary fiber; SDF—soluble dietary fiber.

Figure 2 illustrates the microstructures of IDF and SDF from the four grape varieties. Overall, IDF (Figure 2A–D) exhibited more layered structures, presenting a compact surface with fewer voids. SDF (Figure 2E–H) exhibited more three-dimensional granular structures, with a more varied size of porous cavities. Compared with Kyoho’s insoluble dietary fiber (K-IDF) and Shine Muscat’s insoluble dietary fiber (S-IDF), the microstructures of the Muscadine grape Alachua’s insoluble dietary fiber (A-IDF) and Granny Val’s insoluble dietary fiber (G-IDF) were similar and were flatter and more compact.

#### 2.2.3. X-ray Diffraction

**Figure 3:**
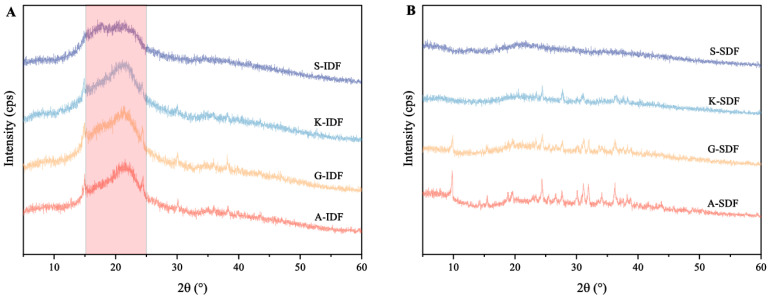
Differences in X-ray diffraction (XRD) patterns of IDF and SDF from four grape varieties. (**A**) XRD of IDF from Shine Muscat (S), Kyoho (K), Alachua (A), and Granny Val (GV). (**B**) XRD of SDF from Shine Muscat (S), Kyoho (K), Alachua (A), and Granny Val (GV). IDF—insoluble dietary fiber; SDF—soluble dietary fiber.

X-ray diffraction was used for the structural determination of crystalline polysaccharides (Figure 3). The IDFs from the four grape varieties showed a passivation diffraction when 2θ ranged between 15° and 25°, indicating the presence of an amorphous or semi-crystalline structure (Figure 3A). There were no pronounced diffraction peaks within 5°~24°, suggesting that the SDFs in the four grape varieties types mainly existed in an amorphous form (Figure 3B).

#### 2.2.4. Functional Groups of IDF and SDF

**Figure 4:**
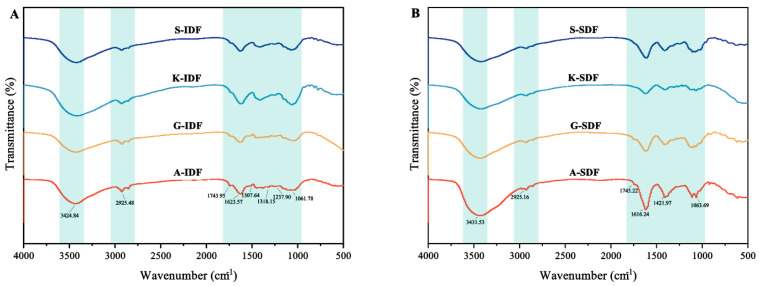
FTIR spectra of IDF and SDF from four grape varieties. (**A**) FTIR spectra of IDF from Shine Muscat (S), Kyoho (K), Alachua (A), and Granny Val (GV). (**B**) FTIR spectra of SDF from Shine Muscat (S), Kyoho (K), Alachua (A), and Granny Val (GV). IDF—insoluble dietary fiber; SDF—soluble dietary fiber.

Functional groups of IDF and SDF were analyzed with infrared spectroscopy (Figure 4). The transitions of SDF and IDF from the four grape varieties were observed at 3424 cm^−1^ and 3431 cm^−1^, respectively, indicating the presence of O–H. Both SDF and IDF had absorption peaks at 2925 cm^−1^, representing the primary vibrations from the methylene groups in sugars [26], indicating typical cellulose structures. All IDFs and SDFs from Granny Val and Alachua exhibited a minor peak around 1745 cm^−1^, indicating the presence of uronic acid in the studied DF. The peak around 1620 cm^−1^ in SDF was attributed to a C=O asymmetric stretching vibration of the carboxyl group. The absorption peak around 1060 cm^−1^ originated from the C–O bond.

### 2.3. Functional Properties

#### 2.3.1. Water Holding, Water Swelling, and Oil Holding Capacities

**Figure 5:**
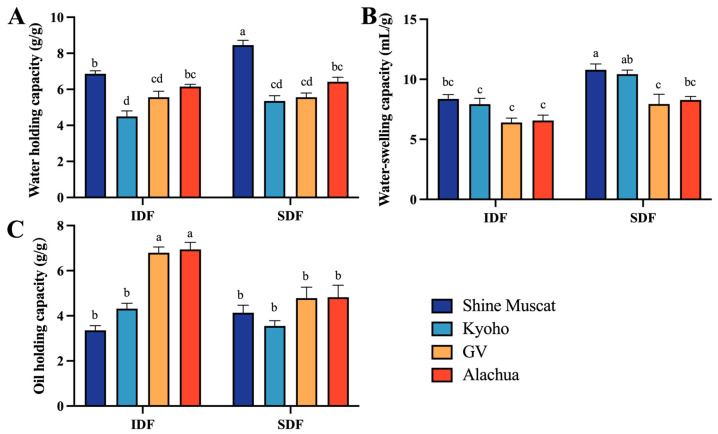
Physical and functionality properties of IDF and SDF from four grape varieties. (**A**) Comparison of water holding capacity between IDF (left) and SDF (right) in four grape varieties. (**B**) Comparison of water swelling capacity between IDF (left) and SDF (right) in four grape varieties. (**C**) Comparison of oil holding capacity between IDF (left) and SDF (right) in four grape varieties. IDF—insoluble dietary fiber; SDF—soluble dietary fiber. a,b,c,d—different small letters indicate significant difference (*p* < 0.05).

The water holding capacity (WHC) of IDF ranged from 4.49 (Kyoho) to 6.86 g/g (Shine Muscat), while that of SDF ranged from 5.35 (Kyoho) to 8.45 g/g (Shine Muscat). Among all studied DFs, the SDF in Shine Muscat had the highest WHC. The IDF in Kyoho had the lowest WHC (Figure 5A). This may be due to the presence of more amorphous cellulose structures in IDF in Shine Muscat, where free hydroxyl groups that have not formed hydrogen bonds exist. Notably, the WHC of SDF in the Shine Muscat grape exceeded that in the other grapes.

The water swelling capacity (WSC) of IDF ranged from 6.41 (Granny Val) to 8.36 mL/g (Shine Muscat), while that of SDF ranged from 7.95 (Granny Val) to10.79 mL/g (Shine Muscat). Among all studied DFs, the SDF in Shine Muscat had the highest WSC. The IDF in Granny Val had the lowest WHC (Figure 5B).

The oil holding capacity (OHC) of IDF ranged from 3.36 (Shine Muscat) to 6.95 g/g (Alachua). The IDF in Alachua (6.95 g/g) and Granny Val A-IDF (6.80 g/g) exhibited significantly higher oil adsorption capabilities than the counterpart conventional table grapes. The OHC of SDF ranged from 3.55 (Kyoho) and 4.82 g/g (Alachua). Among all studied DFs, the IDF from Alachua had the highest OHC. The IDF from Shine Muscat had the lowest OHC (Figure 5C).

#### 2.3.2. Cholesterol and Heavy Metal Adsorption Capacity

**Figure 6:**
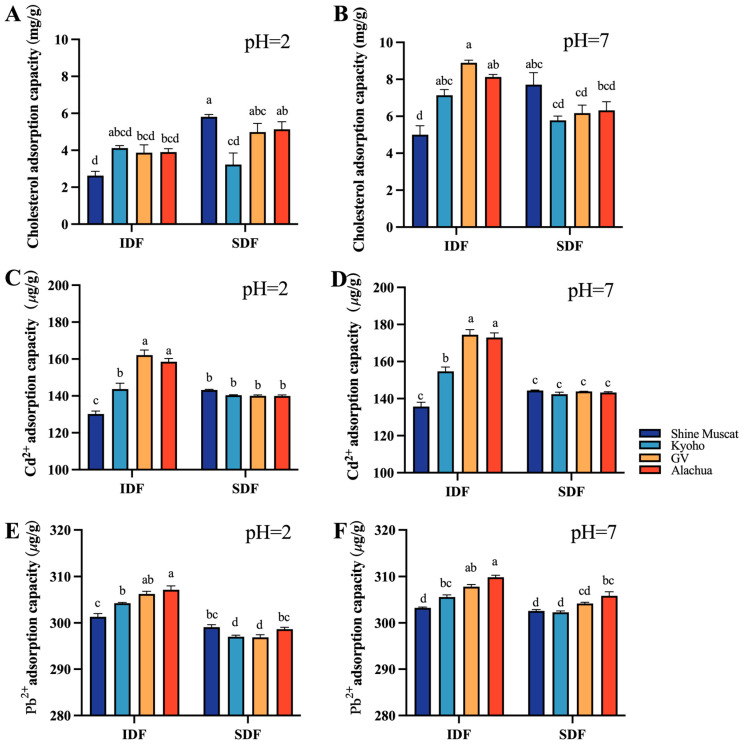
Cholesterol adsorption capacity and heavy metal adsorption capacity at different pHs. Comparison of cholesterol absorption capacity between IDF (left) and SDF (right) from four grape varieties at pH 2 (**A**) and at pH 7 (**B**). Comparison of Cd 2+ absorption capacity between IDF (left) and SDF (right) from four grape varieties at pH 2 (**C**) and at pH 7 (**D**). Comparison of Pb^2+^ absorption capacity between IDF (left) and SDF (right) from four grape varieties at pH 2 (**E**) and at pH 7 (**F**). IDF—insoluble dietary fiber; SDF—soluble dietary fiber. a,b,c,d—different small letters indicate significant difference (*p* < 0.05).

The cholesterol adsorption capacity (CAC) of all studied DFs (including IDF and SDF) were lower at pH 2 (Figure 6A) compared with their counterpart at pH 7 (Figure 6B). At pH 2, the SDF from Shine Muscat had the highest CAC. At pH7, the IDF from Granny Val had the highest CAC. Among all studied DFs, the IDF from Shine Muscat had the lowest CAC at pH 2 and pH 7.

The heavy metal adsorption capacity (HMAC) included the cadmium absorption capacity (Figure 6C,D) and lead absorption capacity (Figure 6E,F) in the current study. The heavy metal absorption capacity of IDF was mostly higher than its SDF counterpart. The cadmium absorption capacity of IDF from Alachua and Granny Val was significantly better than that of IDF from Kyoho and Shine Muscat at pH 2 and pH 7 (Figure 6C,D). No significant difference in the cadmium absorption capacity was identified among SDFs from the four grape varieties at pH 2 and at pH 7 (Figure 6C,D). The IDF lead absorption capacity was highest in Alachua, followed by Granny Van, Kyoho, and Shine Muscat, both at pH 2 and pH 7 (Figure 6E,F). The SDF lead absorption capacity was higher at pH 7 compared with pH 2. At pH 2, the SDF lead absorption capacity was significantly higher in Alachua and Shine Muscat than in Granny Val and Kyoho.

#### 2.3.3. Antioxidant Capacity

**Figure 7:**
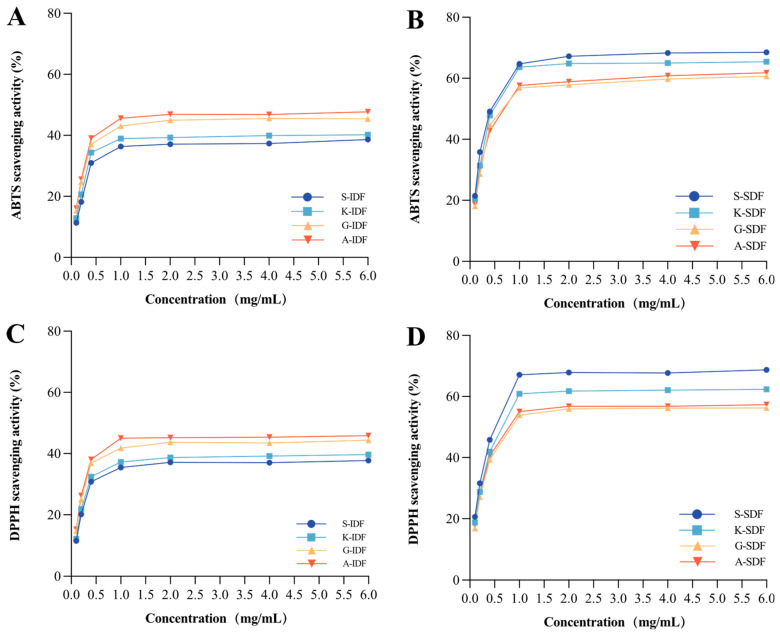
Antioxidant capacity. ABTS scavenging activity of IDF (**A**) and SDF (**B**) in four grape varieties. DPPH scavenging activity of IDF (**C**) and SDF (**D**) in four grape varieties. A-IDF—Alachua insoluble dietary fiber; G-IDF—Granny Val insoluble dietary fiber; K-IDF—Kyoho insoluble dietary fiber; S-IDF—Shine Muscat insoluble dietary fiber; A-SDF—Alachua soluble dietary fiber; G-SDF—Granny Val soluble dietary fiber; K-SDF—Kyoho soluble dietary fiber; S-SDF—Shine Muscat soluble dietary fiber. IDF—insoluble dietary fiber; SDF—soluble dietary fiber.

The antioxidant capacities of IDF and SDF from different grapes were evaluated based on ABTS (Figure 7A,B) and DPPH (Figure 7C,D) scavenging assay. The results of ABTS and DPPH concerning the antioxidant capacity of the studied IDF and SDF were in line with each other. Specifically, the ABTS and DPPH scavenging activity of SDF was higher than IDFs at stationary phases. SDF from Shine Muscat had the highest ABTS and DPPH scavenging activity among all studied SDFs in the current study at the stationary phase. IDF from Alachua had the highest ABTS and DPPH scavenging activity among all studied IDFs.

## 3. Discussion

In the current study, we investigated the structural, physiochemical, and functional properties of DF from four grape varieties, namely Shine Muscat, Kyoho, Granny Val, and Alachua. We hypothesized that DF from different grapes differed in their structural, physiochemical, and functional properties. We found TDF (mainly IDF, with a small fraction of SDF) dominated the grape power. Granny Val and Alachua had significantly higher abundances of TDF and IDF compared with Shine Muscat and Kyoho. Shine Muscat had a significantly higher abundance of SDF compared with the other grapes. Despite the differences in grape varieties, TDF was dominated by cellulose, followed by pectin and hemicellulose. The lignin content in Granny Val and Alachua was significantly higher than in Kyoho and Shine Muscat. Among the four grape varieties, the top two monosaccharides within the IDFs were glucose and xylose, while in the SDF fraction they were galacturonic acid, glucose, galactose, and arabinose. Among all studied DFs, the SDF from Shine Muscat had the highest WHC and WSC but the lowest OHC, while the IDF from Alachua had the highest OHC. The heavy metal absorption capacity of IDF was mostly higher than its SDF counterpart. The CAC of all studied DFs varied at different pHs (mostly values at pH 7 > values at pH 2). At pH 7, the IDF from Granny Val and Alachua not only had the highest CAC but also had the highest cadmium and lead absorption capacity. The antioxidant capacity of SDF was higher than its counterpart IDF at the stationary phase. At the stationary phase, the SDF from Shine Muscat had the highest antioxidant activity among all SDFs, while the IDF from Alachua had the highest antioxidant activity among all studied IDFs.

Being the key component of TDF, cellulose is a glucose-made unbranched polymeric molecule. Hemicellulos also contains glucose units but it is a branched polymer with xylose, mannose, arabinose, galactose, and glucuronic acid [23]. Spinei et al. [24] indicated that grape skin structures are enriched by 25% to 50% hemicellulose, predominantly composed of xyloglucan, which is constructed on a β(1→4)-linked glucan backbone with a branching of 75% glucose and xylose and 35% galactose [27]. The monosaccharide profiles of the four grape varieties in this study, with high proportions of glucose and xylose, corroborate the hemicellulosic component of IDF being rich in xyloglucans. Therefore, the preeminent proportion of glucose in the monosaccharide analysis of these grapes could be attributed on the one hand to the hydrolytic products of cellulose, that is glucose, and on the other hand to the hydrolysis of xyloglucans, a hemicellulosic substance, yielding a great abundance of glucose.

In this study, the SDF from the four grape varieties showed significantly higher contents of galacturonic acid, arabinose, rhamnose, and galactose, which are typical constituents of pectin [28]. Among these, the prominent amount of galacturonic acid in the SDF of the four grape varieties was due to the presence of homogalacturonan (HG), a pectin structural domain identified in grape skin [24], with galacturonic acid being the main structural unit of HG [29]. The SDF from Shine Muscat was particularly rich in arabinose, which has been reported to have physiological activities such as regulating lipid metabolism and influencing intestinal microbiota and metabolism [30,31]. Additionally, the relatively higher content of rhamnose in the SDF from Shine Muscat suggests that it may contain a greater portion of pectin [16].

The porosity and regional chemical properties of the studied DFs can explain some of their physiological effects (like the adsorption and/or binding of certain molecules) [32]. Compared with the configuration of IDF, SDF exhibited more three-dimensional granular structures with more varied sizes of porous cavities, which provided a larger surface area [33]. Findings by Chen et al. [34] revealed that wheat SDF possesses a relatively flat and loose structure with certain gaps between fibers, while the surface of wheat IDF is irregular, characterized by many cracks and small clumps. Similar results were also obtained by Li et al. [35]. A larger specific surface area could allow more space to store water molecules through hydrogen bonding and/or dipole formation [36,37]. This explains the observation (SDFs demonstrated greater capacity in water holding compared with their IDF counterparts) in the current study. Moreover, DFs absorb water molecules via hydrogen bonds and dipole interactions [38] or encase them within their microstructures. Among all studied DFs, the SDF from Shine Muscat had the highest water holding capacity. This can be attributed to its porous nature, which exposes more uronic acid structures and has a larger surface area, enhancing its water retention capability [39]. Furthermore, porous and folded structures can increase the specific surface area and expose more polar groups, consequently promoting the adsorption and binding of water and aiding in its application in foods [40].

DFs adsorb heavy metal ions through both physical and (predominantly) chemical mechanisms [41], involving interactions with functional groups like hydroxyl (–OH), carboxyl (–COOH), carbonyl (C=O), and amino (–NH2) via chelation, complexation, and ion exchange and/or absorption [42]. Within the current study, the heavy metal absorption capacity of IDF was mostly higher than its SDF counterpart. This could in part be attributed to the greater peak at 1620 cm^−1^ in SDF, indicating the presence of a C=O asymmetric stretching vibration of the carboxyl group.

The DF oil holding capacity (OHC) primarily correlates with the specific surface area, surface activity, porosity, total charge density, hydrophobic groups, and capillary forces [43,44,45]. Among all studied DFs, the IDF from Shine Muscat had the lowest OHC. Similarly, among all studied DFs, the IDF from Shine Muscat had the lowest cholesterol adsorption capacity (CAC) at pH 2 and pH 7. Nevertheless, two mechanisms exist for DF cholesterol adsorption, namely physical and chemical adsorption. Specifically, structural properties, such as particle size, porosity, and surface area, of DFs are related to physical adsorption [46,47]. The charges and hydrophobic groups of DFs relate to chemical adsorption. The CAC of all studied DFs (including IDF and SDF) was lower at pH 2 compared with at pH 7. This might be due to the fact that, in acidic environments, both DFs and cholesterol carry positive charges, leading to repulsion and reduced adsorption capabilities [48].

## 4. Materials and Methods

### 4.1. Materials and Reagents

Four grape varieties were included in the current study (Table 2). Specifically, two conventional table grapes (Vitis vinifera), i.e., Shine Muscat and Kyoho, were purchased from the Shi Quan Grape Cooperative Garden (Jinshan District, Shanghai). Two Muscadine grapes (Vitis rotundifolia), i.e., Alachua and Granny Val, were obtained from the Grape Resources Garden of Shanghai Jiao Tong University (School of Agriculture and Biology).

Briefly, in this study, 15 kg of each grape variety was utilized for analysis. The samples were collected in two separate sessions. Granny Val and Alachua varieties were harvested on 21 July, whereas Shine Muscat and Kyoho varieties were collected on 28 July. The four grape varieties for this study were obtained from vineyards in Shanghai’s Jinshan and Minhang districts, both of which experience a subtropical monsoon climate. Jinshan District features hot humid summers and mild moist winters, with an average annual temperature of 16 °C and rainfall of 1200 mm, mostly during the summer. Similarly, Minhang District also maintains a subtropical climate with ample sunlight and moderate rainfall. Both districts adopt irrigation and fertilization strategies that are responsive to local climatic conditions, ensuring favorable growth conditions for grapes.

Cellulase (50 U/mg) and high-temperature alpha-amylase (20,000 U/mL) were purchased from (Qiao Yi Biotechnologies, Shanghai, China). Neutral protease (≥14,000 U/g) was acquired from (Enamar Biotechnology, Shanghai, China). Hexyl hydride, ammonium acetate, and methanol were ordered from (Aladdin Biochemical Technology, Shanghai, China). Absolute ethanol was purchased from (Meryer Chemical Technology, Shanghai, China), and sodium borohydride was purchased from (Sinopharm Chemical Reagent, Shanghai, China). All chemicals used in the current study were of analytical levels.

### 4.2. Proximate Analysis

After manually squeezing out the grape juice, the remaining residues were dried at 65 °C for 18 h (Tianjin Test Instruments, Tianjin, China). Afterwards, they were ground to pass the 60-mesh sieve, which resulted in crude grape powder (2500C, Hong Taiyang Electromechanical, Dongguan, China). The obtained crude grape powder was divided into three portions to be used for the following:

(1) Fundamental constituent analysis.
(2) Separation of DF into pectin, hemicellulose, lignin, and cellulose.
(3) Fraction of DF based on their solubility.

The fundamental constituents of the samples were quantified in accordance with the methodologies prescribed by the Association of Official Analytical Chemists (AOAC). Specifically, the quantification of ash, moisture, crude protein, and fat adhered to the AOAC protocols numbered 942.05, 925.09, 955.04, and 920.39, respectively.

### 4.3. Determination of Pectin, Hemicellulose, Lignin, and Cellulose

The crude grape powder was separated into pectin, hemicellulose, lignin, and cellulose [49]. Briefly, every 1 g of crude powder was mixed with 15 mL 0.5% ammonium acetate (Aladdin Biochemical Technology, Shanghai, China), followed by 1.5 h heating at 90 °C. After cooling to room temperature, the mixed solution was filtered through a 60 mm Buchner filter by a vacuum filtration apparatus (KM-25S, Kemai Instrument, Ningbo, China) and washed with distilled water, pure methanol (Aladdin Biochemical Technology, Shanghai, China), and pure acetone (Sinopharm Chemical Reagent, Shanghai, China) sequentially (2–3 times each). The remaining substance on the filter was then dried and weighed as W1. The filtrate was mixed with anhydrous ethanol (1: 4 *v*/*v*) before centrifugation (Avanti JXN-26, Beckman, Brea, CA, USA) at 6000 rpm 20 min. The obtained pellet was then dried (75 °C for 12 h) and weighed as the pectin substance C1. Residue W1 was mixed with 20 mL of 7% potassium hydroxide (Sinopharm Chemical Reagent, Shanghai, China) containing 0.1% sodium borohydride (Sinopharm Chemical Reagent, Shanghai, China), followed by heating at 85 °C 1.5 h, cooling to room temperature, and filtering (as previously mentioned) to obtain the residue. This residue was then sequentially washed with distilled water, methanol, and acetone, and dried, weighed, and noted as W_2_. The filtrate was neutralized with pure acetic acid (Sinopharm Chemical Reagent, Shanghai, China) before mixing with pure anhydrous ethanol (Meryer Chemical Technology, Shanghai, China) (1: 4 *v*/*v*) for precipitation. After centrifugation at 6000 rpm for 20 min, the pellet was dried (75 °C for 12 h), weighed, and noted as hemicellulose substance C2. The residue substance W_2_ was mixed with 5 mL of 50% sulfuric acid and stored at 4 °C for 12 h, before being filtered, dried, and weighted as W_3_. The ash content in W_3_ was noted as W_4_.

(1) $$Ligin=W_{3}-W_{4}$$

(2) $$Cellulose=W_{2}-W_{3}-W_{4}$$

### 4.4. Separation of Dietary Fibers (DFs) Based on Their Solubility

**Figure 8:**
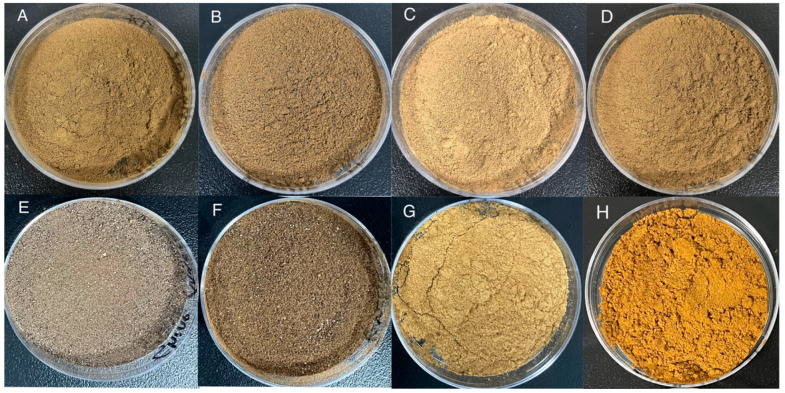
IDF and SDF from four grape varieties. IDF from (**A**) Shine Muscat, (**B**) Kyoho, (**C**) Granny Val, and (**D**) Alachua. SDF from (**E**) Shine Muscat, (**F**) Kyoho, (**G**) Granny Val, and (**H**) Alachua. IDF—insoluble dietary fiber; SDF—soluble dietary fiber.

The crude grape powder was separated into soluble dietary fiber (SDF) and insoluble dietary fiber (IDF) according to the method described earlier [50]. Specifically, crude grape powder was mixed with hexane (2:5 weight (g)/volume (mL)) and heated at 55 °C 90 rpm/min for 2 h (SHA C, Bona Technology, Hangzhou, China). After precipitation, the remaining pellet was dried in a fume hood and mixed with hexane two more times to remove remaining fats. The obtained pellet was mixed with distilled water (1:10 weight (g)/volume (mL)) and the solution pH was adjusted to 7.0 before heating to 90 °C. While keeping the temperature at 90 °C, the obtained solution was treated with 20 mL of high temperature alpha amylase (20,000 U/mL) for 1 h before adjusting the pH to 6.0 at room temperature. The working solution was then heated to 50 °C and treated with 25 mL of protease (≥14,000 U/g) for 4 h before adjusting the pH to 5.0 at room temperature. Afterwards, the working solution was heated to 60 °C and treated by 1.2% cellulase for 2 h. Added enzymes were then deactivated by heating the working solution at 90 °C for 5 min, followed by centrifugation at 4000 rpm/min for 30 min. The obtained pellet was dried at 65 °C overnight as IDF (Figure 8A–D). The obtained supernatant was mixed with 100% ethanol (1:4 *v*/*v*). After precipitation, the obtained pellet was dried at 65 °C overnight as SDF (Figure 8E–H).

### 4.5. Physiochemical Properties of IDF and SDF

#### 4.5.1. Monosaccharide Composition

The monosaccharide composition of isolated SDF and IDF was then analyzed using high performance anion exchange chromatography (HPAEC, ICS-5000+/900, Thermo Fisher, Waltham, MA, USA). Briefly, SDF or IDF (5 mg each) was added to 4 mL of 2 mol/L trifluoroacetic acid and hydrolyzed in a constant temperature oven at 110 °C for 4 h. After cooling, the hydrolysate was filtered through a 0.22 μm filter membrane, and then diluted to 100 μg/mL before analysis. With the analytical method referenced by Wang [51], the chromatographic conditions were as follows: Dionex Carbopac PA100 column with an amperometric detector; Au electrode; NaOH as eluent; flow rate of 0.4 mL/min; injection volume of 10 μL; and column temperature set at 30 °C.

#### 4.5.2. Scanning Electron Microscopy (SEM)

The morphology of the IDF and SDF from the four grape varieties was examined using a scanning electron microscope (S3400II, Hitachi Limited, Tokyo, Japan). Briefly, SDF or IDF was fixed on a sample stage using double-sided conductive tape. The sample surface was sputter-coated with gold using a vacuum gold sputter coater (SCD050, Leica, Germany) at the sputtering site. The SEM images were made at magnifications of 500× and 5000×.

#### 4.5.3. X-ray Diffraction (XRD)

IDF and SDF were set in the holder of the X-ray diffractometer and analyzed using the X-ray diffractometer (D8 ADVANCE Da Vinci, BRUKER, Rheinstetten, Germany) at room temperature. Cu Ka radiation was used, with a voltage of 40 kV and a current of 40 mA. The scanning range was 5–70°, with a scanning speed 5°/min and a step size of 0.02°.

#### 4.5.4. Fourier Transform Infrared (FTIR)

FTIR was determined using a spectrophotometer (Nicolet 6700, Thermo Fisher, Waltham, MA, USA). Briefly, every 1 mg SDF or IDF was mixed with 100 mg potassium bromide (spectroscopic grade). It was then ground in an agate mortar to a homogeneous mixture. The obtained mixture was then compressed into a 1 mm thick tablet. Spectra were recorded in the infrared spectral range of 500–4000 cm^−1^, with 32 scans at a resolution of 4 cm^−1^ at 25 °C.

### 4.6. Functional Properties of IDF and SDF

#### 4.6.1. Water Holding Capacity (WHC)

Every 1.00 g of IDF and SDF (m_1_) was mixed with 20 mL distilled water, which was left to stand for 12 h before mixing with 80 mL anhydrous ethanol. The mixture was set still for 4 h, before centrifugation at 10,000 rpm/min for 10 min. The remaining pellets were weighed (m_2_) and used to calculate the WHC, according to the formula below.

(3) $$\mathrm{WHC}\;(g/g)=\frac{m_{2}\;-\;m_{1}}{m_{1}}$$

#### 4.6.2. Water Swelling Capacity (WSC)

Every 2.00 g of IDF and SDF was placed in graduated test tubes. The initial volume of the sample was recorded (V_1_). After adding 20 mL of distilled water and allowing it to swell at room temperature for 24 h, 80 mL of anhydrous ethanol was added before centrifugation at 10,000 rpm/min for 10 min. The final volume was noted as V_2_.

(4) $$\mathrm{WSC}\;(\mathrm{mL}/g)=\frac{V_{2}-V_{1}}{m}$$

where V_1_ is the volume of the sample before swelling (mL), V_2_ is the volume after swelling (mL), and m is the dry weight of the sample (g).

#### 4.6.3. Oil Holding Capacity (OHC)

Every 0.5 g of IDF and SDF was mixed with 10 g of peanut oil, and was set still at room temperature for 18 h before centrifugation at 4000 rpm/min for 20 min. After removing the remaining oil, the content was reweighted as W_2_.

(5) $$\mathrm{OHC}\;(g/g)=\frac{W_{2}-W_{1}}{W_{1}}$$

where W_1_ represents the dry weight of the sample (g) and W_2_ is the weight of the sample after fat adsorption (g).

#### 4.6.4. Cholesterol Adsorption Capacity (CAC)

Every 0.5 g of IDF and SDF was added to 30 mL of yolk distilled water mixture (1:10 *v*/*v*). The pH of the solution was adjusted to 2.0 and 7.0 and agitated for 2 h. This was followed by centrifugation at 2000 rpm for 15 min. A 1 mL aliquot of the supernatant was taken, diluted five times with 90% ice cold acetic acid, and the absorbance at 550 nm was measured.

(6) $$\mathrm{CAC}\;(\mathrm{mg}/g)=\frac{W_{1}-W_{2}}{W_{0}}$$

where W_1_ is the cholesterol content in yolk solution before adsorption (mg), W_2_ is the cholesterol content in the supernatant after adsorption (mg), and W_0_ is the weight of the sample.

#### 4.6.5. Heavy Metal Adsorption Capacity (HMAC)

The heavy metal adsorption capacity (HMAC) included the cadmium absorption capacity and lead absorption capacity. A sample (1.00 g) was placed in a centrifuge tube. Then, 30 mL of a 50 µmol/L solution of either cadmium nitrate or lead nitrate was added. The pH was adjusted to 2.0 and 7.0 using nitric acid or sodium hydroxide. This mixture was then agitated at 37 °C for 4 h, centrifuged at 5000 rpm for 10 min, and the supernatant was analyzed for lead and cadmium ion concentrations using atomic absorption spectroscopy (AAS).

(7) $$\mathrm{HMAC}\;(\mu g/g)=\frac{(C_{1}-C_{2})\;\times \;V}{W_{0}}$$

where C_1_ is the initial concentration of heavy metal ions in the solution (µg/mL), C_2_ is the concentration of heavy metal ions in the supernatant after adsorption (µg/mL), V is the volume of the sample solution (L), and W_0_ is the weight of the sample (g).

#### 4.6.6. Antioxidant Properties

ABTS (3-ethylbenzothiazoline-6-sulfonic acid) scavenging assay.

The working solution of SDF and IDF was made to 0.1, 0.2, 0.4, 1, 2, 4, and 6 mg/mL with distilled water. The ABTS solution (0.384 g ABTS in 100 mL distilled water) was mixed with equal volume 2.45 mmol/L of K_2_S_2_O_8_ solution and stored in the dark for 16 h to produce ABTS radical cation. Then, the mixture was diluted by 80% of ethanol solution, allowing the absorbance at 734 nm to be 0.70 ± 0.02. The premade SDF and IDF solution was then mixed with the ABTS solution for 10 min at 37 °C, before measuring at 734 nm.

(8) $$\mathrm{ATBS}\;\mathrm{clearance}\;\mathrm{rate}\left(\%\right)=\frac{A_{0}-A}{A_{0}}\times 100\%$$

where A_0_ is the absorbance of the blank and Ac is the absorbance of the sample.

DPPH (2,2-Diphenyl-1-picrylhydrazyl) scavenging assay.

The working solution of SDF and IDF was made to 0.1, 0.2, 0.4, 1, 2, 4, and 6 mg/mL with distilled water. The obtained solution was then mixed with the DPPH solution (4.5 mg DPPH in 100 mL methanol) and kept in the dark for 30 min before measuring at 517 nm.

(9) $$\mathrm{DPPH}\;\mathrm{clearance}\;\mathrm{rate}\left(\%\right)=\frac{A_{c}-(A_{S}-A_{0})}{A_{c}}\times 100\%$$

where Ac is the absorbance of the positive control, A_S_ is the absorbance of the sample, and A_0_ is the absorbance of the blank.

### 4.7. Statistical Analysis

The current study employed a completely randomized design (CRD) to evaluate the dietary fiber content, structural properties, and chemical characteristics of four different grape varieties. In addition, the experimental units were randomly assigned to the different treatments to eliminate systematic errors and ensure that all varieties were subjected to uniform and randomized experimental conditions. Moreover, each test for every grape variety was replicated three times to ensure the reliability and repeatability of the experimental results. Data were organized using Excel 2021. All experiments were performed in triplicate, with results presented as mean ± standard error. One way analysis of variance (ANOVA) was conducted using SPSS 26.0, with post hoc comparisons using the Tukey method. *p* values < 0.05 were considered of significant difference. Graphs were generated using Origin2022 and/or Graphpad Prism 9.3.1.

## 5. Conclusions

In this study, the composition and functional properties of DF from four grape varieties were characterized. The results showed that the IDF content of Muscadine grape (Granny Val and Alachua) was higher than that of conventional table grape (Shine Muscat and Kyoho) IDF, and the structure of Muscadine grape IDF was more compact and flatter. Granny Val and Alachua had a significantly higher abundance of TDF (mainly IDF, with a small fraction of SDF) compared with Shine Muscat and Kyoho. Specifically, the TDF was dominated by cellulose, followed by pectin and hemicellulose. The lignin content of Granny Val and Alachua was significantly higher than of Kyoho and Shine Muscat. Among the four grape varieties, the top two monosaccharides within IDF were glucose and xylose, while they were galacturonic acid, glucose, galactose, and arabinose in SDF. Moreover, the SDF from Shine Muscat had the highest WHC and WSC but the lowest OHC, while the IDF from Alachua had the highest OHC. The CAC of all studied DFs varied at different pHs (mostly values at pH 7 > values at pH 2). The heavy metal absorption capacity of IDF was mostly higher than its SDF counterpart. In contrast, the antioxidant capacity of SDF was higher than that of its counterpart (i.e., IDF) at the stationary phase.

## Figures and Tables

**Table 1:** Monosaccharide composition of SDF and IDF (%) from four grape varieties.

DF		Rhamnose	Arabinose	Galactose	Glucose	Mannose	Xylose	Galacturonic Acid	Glucuronic Acid
IDF	Shine Muscat	0.31 ± 0.02 b	4.93 ± 0.21 c	1.95 ± 0.02 d	**79.89 ± 0.31 a**	2.14 ± 0.09 b	7.56 ± 0.24 d	2.81 ± 0.15 d	0.41 ± 0.03 c
	Kyoho	0.63 ± 0.1 a	6.38 ± 0.08 ab	9.48 ± 0.32 a	**49.84 ± 0.63 c**	0.98 ± 0.06 c	**25.5 ± 0.25 b**	6.18 ± 0.35 b	1.02 ± 0.08 b
	Granny Val (GV)	0.26 ± 0.04 b	5.5 ± 0.24 bc	3.29 ± 0.24 c	**62.92 ± 0.33 b**	2.64 ± 0.17 b	**19.29 ± 0.19 c**	5.18 ± 0.12 c	0.93 ± 0.12 b
	Alachua	0.09 ± 0.01 b	7.23 ± 0.22 a	6.39 ± 0.28 b	**38.89 ± 1 d**	8.68 ± 0.29 a	**28.26 ± 0.73 a**	8.31 ± 0.05 a	2.15 ± 0.13 a
SDF	Shine Muscat	3.59 ± 0.09 a	**18.93 ± 0.3 a**	**16.54 ± 0.56 ab**	**20.74 ± 0.34 b**	0	1.59 ± 0.05 b	**35.4 ± 0.66 a**	3.21 ± 0.12 c
	Kyoho	1.87 ± 0.08 c	**10.23 ± 0.26 c**	**13.22 ± 0.67 b**	**48.41 ± 0.93 a**	1.52 ± 0.07 c	1.36 ± 0.04 b	**20.31 ± 0.06 d**	3.09 ± 0.02 c
	Granny Val (GV)	2.62 ± 0.12 b	**11.52 ± 0.37 bc**	**19.95 ± 0.31 a**	**20.1 ± 0.26 b**	6.8 ± 0.03 b	1.1 ± 0.04 c	**32.02 ± 0.42 b**	5.89 ± 0.14 b
	Alachua	1.87 ± 0.16 c	**12.26 ± 0.56 b**	**13.93 ± 1.6 b**	**21.7 ± 0.1 b**	**14.64 ± 0.35 a**	3 ± 0.08 a	**24.59 ± 0.37 c**	8.02 ± 0.27 a

IDF—insoluble dietary fiber; SDF—soluble dietary fiber. Bold text highlights average relative abundance above 10%. Data are represented as mean ± standard deviation (n = 3). Different small letters within a column indicate statistically significant differences between groups at *p* < 0.05.

**Table 2:** Varieties and growing regions of the four grape varieties.

Varieties		Region
Vitis vinifera	Shine Muscat	Jinshan, Shanghai, China
	Kyoho	Jinshan, Shanghai, China
Vitis rotundifolia	Granny Val (GV)	Minhang, Shanghai, China
	Alachua	Minhang, Shanghai, China

## Data Availability

Data will be made available on request.
